# Non-pulmonary cancer risk following tuberculosis: a nationwide retrospective cohort study in Lithuania

**DOI:** 10.1186/s13027-017-0143-8

**Published:** 2017-05-31

**Authors:** Ruta Everatt, Irena Kuzmickiene, Edita Davidaviciene, Saulius Cicenas

**Affiliations:** 1 Laboratory of Cancer Epidemiology, National Cancer Institute, Baublio g. 3B, LT-08406 Vilnius, Lithuania; 2 Vilnius University Hospital Santaros Klinikos, P. Sirvio g. 5, LT-10214 Vilnius, Lithuania; 3 Department of Thoracic Surgery and Oncology, National Cancer Institute, Santariskiu g. 1, LT-08660 Vilnius, Lithuania; 4 Faculty of Medicine, Vilnius University, M. K. Ciurlionio g. 21, LT-03101 Vilnius, Lithuania

**Keywords:** Non-pulmonary cancers, Risk factors, Tuberculosis, Infection, Retrospective cohort study, Lithuania

## Abstract

**Background:**

Lithuania remains one of the highest tuberculosis burden countries in Europe. Epidemiological studies have long pointed to infections as important factors of cancer aetiology, but the association between tuberculosis and the risk of non-pulmonary cancers has rarely been tested and results have been inconsistent. The aim of this population-based cohort study was to examine the risk of cancer among patients diagnosed with tuberculosis using data from Lithuanian Tuberculosis, Cancer and Resident’s Registries.

**Methods:**

The study cohort included 21,986 tuberculosis patients yielding 1583 cancers diagnosed during follow-up (1998–2012). Standardized incidence ratios (SIRs) and 95% confidence intervals (95% CIs) were calculated to compare the incidence of cancer among cohort participants with the general population for overall, non-pulmonary, site-specific cancers, as well as for subgroups of smoking-related, alcohol-related, hormone-related and haematological cancers.

**Results:**

The SIRs of all cancers combined were 1.89, 95% CI: 1.79–2.00 in men and 1.34, 95% CI: 1.19–1.50 in women. Risk was increased 3-fold within the first year following diagnosis; it decreased during later years, although remained significantly elevated for ≥5 years. Elevated long-term increased risks persisted for non-pulmonary cancers overall, and for cancers of mouth and pharynx, oesophagus, stomach, larynx, cervix uteri and leukaemias. Tuberculosis was associated with a decreased risk of melanoma. Increased risks were observed for smoking-related cancers in men (SIR 1.95, 95% CI: 1.79–2.13) and women (SIR 1.46, 95% CI: 1.22–1.73), alcohol-related cancers in men (SIR 2.40; 95% CI: 2.14–2.68) and haematological cancers in men (SIR 1.73, 95% CI: 1.33–2.23). The risk of hormone-related cancers was 18% lower (SIR = 0.82, 95% CI: 0.66–0.997) among women, the inverse association was weaker among men (SIR = 0.95, 95% CI: 0.84–1.07).

**Conclusions:**

The risk of total and several non-pulmonary cancers was elevated in a cohort of tuberculosis patients. The recommendation for the awareness of this association among physicians is warranted. Analysis suggests a reduction in risk of hormone-related cancers and melanoma.

## Background

The latest World Health Organization (WHO) global tuberculosis report shows 10.4 million new tuberculosis cases and 1.4 million deaths due to tuberculosis worldwide in 2015 [1]. With 1600 new cases (56 cases per 100,000 population) in 2015 Lithuania remains among the countries with the highest incidence of tuberculosis in Europe, although due to recent decrease in rates it is no longer in the WHO’s list of high tuberculosis burden countries [1]. The incidence of cancer of several types in Lithuania is among the highest in the world [2].

In addition to smoking or environmental factors, a growing number of bacterial and parasitic infections have been associated with development of cancer [3]. Former analyses observed an increased risk of lung cancer in tuberculosis cohorts, although conclusions with regards to confounding effects of smoking were inconsistent [4–7]. Recently the association between tuberculosis infection and certain non-pulmonary malignancies (multiple myeloma [8]; myeloid leukaemia [4], leukaemia [9], Non-Hodgkin lymphoma [4, 10, 11], Hodgkin lymphoma [11, 12], melanoma [13], as well as oesophageal [4, 9], kidney and bladder [8, 9], liver [4, 9] and other cancers [8, 9]) has been reported; however, the evidence remains inconclusive due to the low incidence of cancer following tuberculosis, small sample size or short follow-up. Previous studies led to the suggestion that inflammation caused by *Mycobacterium tuberculosis* infection, could induce genetic damage promoting carcinogenesis [14]. Prior tuberculosis might also indicate the immune deficiency and increased risk for cancer as a result. In addition, chronic immune stimulation and certain infectious diseases have been shown to have a protective effect against cancer, although studies have been inconsistent [11, 15, 16]. Detailed investigation of association between tuberculosis and cancer is important for elucidating the role of this chronic infection in occurrence of cancer as well as for developing an effective cancer prevention strategy.

In this analysis, we aimed to investigate association between tuberculosis infection and risk of overall and site-specific non-pulmonary cancer using a population-based national cohort data.

## Methods

### Study population

The study was performed using new cases of tuberculosis diagnosed from January 1, 1998 to December 31, 2012; they were identified at the Lithuanian Tuberculosis registry, which covers the entire population. For each individual, information on date of birth, sex, date of diagnosis, diagnosis codes according to ICD-10 (International Statistical Classification of Diseases, 10th Revision) was obtained. In all, 30,594 individuals were available for analysis. Since the occurrence of cancer is rare in the youngest age groups, and diagnoses of the disease are less reliable for those in old age [17] only individuals of 25 to 75 years old at tuberculosis diagnosis were included. More detailed information about the study has been published elsewhere [7]. We excluded records due to age (19.8%), cancer other than non-melanoma skin cancer before start of follow-up (4.8%), unknown vital status (3.1%), duplicates or insufficient data (0.4%), and not being a Lithuanian citizen (0.03%). The final number of participants, included in the current analysis, was 21,986. For patients with multiple cancers, each non-melanoma skin cancer diagnosis was considered as an individual event.

### Case ascertainment

For each subject, date of entry into cohort was the date of diagnosis of tuberculosis, and date of exit was the date of diagnosis of cancer other than non-melanoma skin cancer, death, emigration, or 31 December 2012, whichever occurred first. We identified cases of cancer from the Lithuanian Cancer Registry (LCR). The LCR has population-based information available since 1978; data since the year 1988 have been included in ‘Cancer Incidence in Five Continents’ [18]. Dates of death and emigration were ascertained by linkage to the Lithuanian Residents’ Register Service. The linkages were performed using an individual personal identification number. For calculation of Standardized incidence ratios (SIRs), cancer incidence and population data were obtained from the LCR and Lithuanian Department of Statistics.

### Data analysis

We calculated SIRs of total and site-specific non-pulmonary cancer in the tuberculosis cohort, based on person-days of ‘follow-up’. Expected numbers were obtained by multiplying Lithuanian national cancer incidence rates according to sex-, age- (5-year groups) and calendar period (1998–2002, 2003–2007 and 2008–2012), by corresponding stratum-specific number of person-days of follow-up accrued in the cohort. Then these stratum-specific expected numbers were summed across all strata in the cohort to obtain the expected total number of people with lung cancer in the cohort. We calculated SIRs by dividing the observed number of events by that expected. We calculated 95% confidence intervals (95% CI) for the SIRs assuming a Poisson distribution. Stratified analysis was performed according to the years since tuberculosis diagnosis and sex. SIRs were computed for total, non-pulmonary, tobacco-related [19], alcohol-related [19], haematological, hormone-related [20, 21] and site-specific cancers.

Analyses were performed using SPSS 19. All tests were two-tailed, and *P* value below 0.05 was considered statistically significant.

## Results

A total of 21,986 patients (70.3% male) with tuberculosis were enrolled in the study (Table 1). The mean age at tuberculosis diagnosis was 47.1 years. The percent of smokers was 63.8% among total population, 78.0% among men and 30.2% among women. Among patients with tuberculosis, 59.3% were unemployed and only 6.4% had high school education. We followed the cohort for a total of 136,816 person-years. During the follow-up we observed 1583 cancers. There were 62 persons having two cancers.

**Table 1:** Characteristics of cohort members

Factors	*n* (%)
Total	21,986 (100)
Sex	
Men	15,456 (70.3)
Women	6530 (29.7)
High school education	1401 (6.4)
Smokers	14,023 (63.8)
Heavy alcohol user	7679 (34.9)
Unemployed	13,038 (59.3)
Age at tuberculosis diagnosis, years, mean (SD)	47.1 (12.9)
Follow-up duration, years, mean (SD)	6.2 (4.4)
Total follow-up duration, person-years	136,816
Cancer cases	1583
Respiratory tuberculosis	20,302 (92.3)
Human immunodeficiency virus infection	77 (0.4)

Total cancer SIR among tuberculosis patients, compared with the general population was 1.76, 95% CI: 1.67–1.85 (Table 2). When lung cancer was excluded, all-time SIR was 1.41, 95% CI: 1.33–1.50. Short-term and long-term risks of non-pulmonary cancer were significantly increased, SIR = 2.03, 95% CI: 1.75–2.34 within first 1 year after diagnosis, 1.38, 95% CI: 1.25–1.52 during 1–4 years and 1.29, 95% CI: 1.18–1.41 during ≥5 years of follow-up (Table 2).

**Table 2:** Cancer incidence in patients with tuberculosis according to time since tuberculosis diagnosis

Type of cancer	ICD-O	Time since tuberculosis diagnosis											
		<1 year			1–4 years			≥5 years			Total		
		Exp	Obs	SIR (95% CI)	Exp	Obs	SIR (95% CI)	Exp	Obs	SIR (95% CI)	Exp	Obs	SIR (95% CI)
All but NMS	C00–96, excl. C44	107.7	347	3.22 (2.89–3.58)	377.0	625	1.74 (1.60–1.88)	450.5	611	1.36 (1.25–1.47)	935.2	1583	1.76 (1.67–1.85)
All but NMS & lung	C00–96, excl. C44, C33, C34	91.6	186	2.03 (1.75–2.34)	310.9	429	1.38 (1.25–1.52)	379.6	490	1.29 (1.18–1.41)	782.1	1105	1.41 (1.33–1.50)
Mouth, pharynx	C01–14	4.3	20	4.65 (2.84–7.18)	13.8	57	4.27 (3.24–5.54)	13.9	45	3.24 (2.36–4.33)	32.0	122	3.92 (3.25–4.68)
Oesophagus	C15	2.1	9	4.28 (1.96–8.13)	7.0	28	4.22 (2.80–6.10)	7.3	26	3.57 (2.33–5.23)	16.4	63	3.99 (3.07–5.11)
Stomach	C16	7.6	16	2.11 (1.21–3.44)	24.9	30	1.28 (0.86–1.83)	27.0	45	1.67 (1.22–2.23)	59.4	91	1.60 (1.29–1.96)
Colon	C18	4.9	2	0.41 (0.05–1.47)	17.4	20	1.21 (0.74–1.87)	22.3	18	0.81 (0.48–1.27)	44.7	40	0.93 (0.67–1.27)
Rectum	C19–21	4.8	6	1.25 (0.46–2.75)	16.7	14	0.89 (0.49–1.48)	20.2	23	1.14 (0.72–1.71)	41.7	43	1.08 (0.78–1.45)
Liver	C22	1.2	3	2.47 (0.51–7.25)	4.3	9	2.20 (1.01–4.19)	5.5	9	1.63 (0.74–3.09)	11.1	21	1.98 (1.23–3.03)
Pancreas	C25	3.3	10	3.00 (1.44–5.52)	11.3	16	1.50 (0.86–2.43)	13.2	16	1.21 (0.69–1.97)	27.8	42	1.57 (1.13–2.12)
Larynx	C32	2.7	14	5.17 (2.82–8.67)	8.7	22	2.67 (1.67–4.04)	8.7	17	1.96 (1.14–3.14)	20.0	53	2.74 (2.05–3.59)
Bone, mesothelial & soft tissue	C40–41, C45, C47, C49	0.9	4	4.43 (1.21–11.40)	2.9	4	1.44 (0.39–3.68)	3.1	6	1.97 (0.72–4.28)	6.8	14	2.11 (1.15–3.54)
Melanoma	C43	1.7	1	0.58 (0.02–3.22)	6.0	3	0.51 (0.11–1.50)	7.3	3	0.41 (0.08–1.20)	15.0	7	0.48 (0.19–0.96)
Breast^a^	C50	5.7	3	0.53 (0.11–1.55)	20.3	15	0.74 (0.41–1.22)	25.9	32	1.21 (0.83–1.70)	51.9	50	0.96 (0.72–1.27)
Cervix uteri	C53	2.4	6	2.51 (0.92–5.44)	8.2	20	2.43 (1.48–3.75)	9.2	25	2.73 (1.77–4.03)	19.7	51	2.60 (1.93–3.42)
Corpus uteri	C54, C55	2.1	5	2.36 (0.77–5.50)	7.5	2	0.26 (0.03–0.95)	10.3	6	0.58 (0.21–1.27)	19.9	13	0.66 (0.35–1.13)
Ovary	C56	1.7	5	2.95 (0.96–6.86)	5.8	2	0.34 (0.04–1.23)	7.4	9	1.22 (0.56–2.32)	14.9	16	1.08 (0.62–1.76)
Prostate	C61	20.9	16	0.76 (0.44–1.24)	84.0	66	0.83 (0.64–1.05)	114.2	102	0.89 (0.73–1.08)	219.2	184	0.88 (0.76–1.02)
Kidney	C64	5.6	12	2.13 (1.10–3.72)	19.0	20	1.10 (0.67–1.70)	21.2	14	0.66 (0.36–1.11)	45.9	46	1.04 (0.76–1.38)
Bladder	C67	3.5	14	3.95 (2.16–6.62)	11.7	19	1.77 (1.06–2.76)	13.3	15	1.13 (0.63–1.87)	28.5	48	1.79 (1.32–2.37)
Brain, CNS	C70–72	2.1	3	1.45 (0.30–4.26)	6.8	9	1.36 (0.62–2.59)	7.3	5	0.69 (0.22–1.60)	16.1	17	1.08 (0.63–1.73)
Thyroid	C73	1.8	1	0.57 (0.01–3.15)	6.3	2	0.32 (0.04–1.16)	7.6	7	0.92 (0.37–1.90)	15.6	10	0.65 (0.31–1.19)
HL	C81	0.5	2	4.13 (0.50–14.90)	1.4	4	2.83 (0.77–7.25)	1.2	2	1.74 (0.21–6.30)	3.1	8	2.65 (1.14–5.22)
NHL & other LHT	C82–85, C88, C90, C96	3.0	8	2.71 (1.17–5.34)	10.6	15	1.46 (0.82–2.41)	13.2	11	0.84 (0.42–1.49)	26.7	34	1.31 (0.91–1.84)
Leukaemias	C91–95	2.6	3	1.16 (0.24–3.39)	8.8	18	2.14 (1.27–3.38)	10.6	20	1.90 (1.16–2.93)	22.0	41	1.94 (1.39–2.63)
Other		6.0	23	3.84 (2.43–5.76)	19.5	34	1.75 (1.21–2.44)	22.7	34	1.50 (1.04–2.10)	48.1	91	1.79 (1.43–2.21)

*Exp* expected cases, *Obs* observed cases, *SIR* standardized incidence ratio, *CI* confidence interval, *NMS* non-melanoma skin, *HL* Hodgkin lymphoma, *NHL* Non-Hodgkin lymphoma, *LHT* lympho-, haematopoietic tissue

^a^in women only, in men *n* = 0

Analysis by cancer type revealed high risks of mouth and pharynx, oesophagus, stomach, liver, pancreas, larynx and bladder cancer (Table 2). Tuberculosis was also positively associated with leukaemias, Hodgkin lymphoma, bone, mesothelial & soft tissue as well as “other” cancers. In addition, results revealed reduced risk of melanoma, SIR = 0.48, 95% CI: 0.19–0.96. Risk of most non-pulmonary cancers was significantly increased within the first year. Elevated long-term risks (≥5 years) persisted for cancers of the mouth and pharynx (SIR = 3.24, 95% CI: 2.36–4.33), oesophagus (SIR = 3.57, 95% CI: 2.33–5.23), stomach (SIR = 1.67, 95% CI: 1.22–2.23), larynx (SIR = 1.96, 95% CI: 1.14–3.14), cervix uteri (SIR = 2.73, 95% CI: 1.77–4.03), leukaemias (SIR = 1.90, 95% CI: 1.16–2.93) and for “other” cancers (SIR = 1.50, 95% CI: 1.04–2.10).

The SIRs of total and most site-specific cancers were higher in men than in women (Table 3). Among men with tuberculosis SIR’s were substantially increased for mouth and pharynx, oesophagus, stomach, liver, larynx, bladder, bone, mesothelial and soft tissue cancer, Hodgkin lymphoma, leukaemias and “other” cancer. In contrast, there was reduced incidence of prostate cancer, SIR 0.88, 95% CI: 0.76–1.02. Women were at increased risk of cancer of mouth and pharynx, larynx and cervix uteri, and at reduced risk of cancer of colon, SIR 0.39, 95% CI: 0.13–0.92. High risks of tobacco-related cancers were observed among men (SIR = 1.95, 95% CI: 1.79–2.13) and women (SIR = 1.46, 95% CI: 1.22–1.73). Alcohol-related cancers showed an increased SIR of 2.40, 95% CI: 2.14–2.68 among men, whereas among women there was no increase in risk. An elevated SIR of haematological cancers was also seen for men (SIR = 1.73, 95% CI: 1.33–2.23), it showed a not statistically significant increase in women (SIR = 1.48, 95% CI: 0.93–2.24). The risk of hormone-related cancers was 18% lower (SIR = 0.82, 95% CI: 0.66–0.997) among women, the inverse association was weaker among men (SIR = 0.95, 95% CI: 0.84–1.07).

**Table 3:** Cancer incidence in patients with tuberculosis according to type, sex and aetiology

Type of cancer	ICD-O	Men			Women		
		Exp	Obs	SIR (95% CI)	Exp	Obs	SIR (95% CI)
All but NMS	C00–96, excl. C44	710.6	1285	1.89 (1.79–2.00)	224.7	298	1.34 (1.19–1.50)
All but NMS & lung	C00–96, excl. C44, C33–34	567.4	850	1.50 (1.40–1.60)	214.6	255	1.19 (1.05–1.34)
Mouth, pharynx	C01–14	30.0	116	3.98 (3.28–4.77)	2.0	6	3.05 (1.12–6.64)
Oesophagus	C15	15.5	60	4.01 (3.06–5.16)	0.8	3	3.72 (0.77–10.90)
Stomach	C16	47.8	75	1.65 (1.30–2.07)	11.7	16	1.38 (0.79–2.24)
Colon	C18	31.8	35	1.16 (0.81–1.62)	12.9	5	0.39 (0.13–0.92)
Rectum	C19–21	31.8	37	1.23 (0.87–1.70)	10.0	6	0.61 (0.22–1.32)
Liver	C22	9.0	19	2.23 (1.34–3.48)	2.1	2	0.98 (0.12–3.52)
Pancreas	C25	21.6	33	1.60 (1.10–2.25)	6.2	9	1.47 (0.67–2.79)
Larynx	C32	19.7	49	2.58 (1.91–3.42)	0.4	4	10.88 (2.95–27.7)
Bone, mesothelial & soft tissue	C40–41, C45, C47, C49	5.0	11	2.29 (1.14–4.09)	1.8	3	1.64 (0.34–4.82)
Melanoma	C43	8.9	4	0.46 (0.13–1.19)	6.1	3	0.50 (0.10–1.45)
Breast	C50		-		51.9	50	0.96 (0.72–1.27)
Cervix uteri	C53		-		19.7	51	2.60 (1.93–3.42)
Corpus uteri	C54, C55		-		19.9	13	0.66 (0.35–1.13)
Ovary	C56		-		14.9	16	1.08 (0.62–1.76)
Prostate	C61	219.2	184	0.88 (0.76–1.02)	-		
Kidney	C64	36.8	34	0.96 (0.67–1.34)	9.1	12	1.34 (0.69–2.33)
Bladder	C67	25.6	44	1.83 (1.33–2.46)	2.9	4	1.39 (0.38–3.57)
Brain, CNS	C70–72	11.3	15	1.37 (0.76–2.25)	4.8	2	0.42 (0.05–1.52)
Thyroid	C73	4.8	2	0.43 (0.05–1.55)	10.9	8	0.74 (0.32–1.47)
HL	C81	2.0	6	2.98 (1.09–6.49)	1.0	2	1.98 (0.24–7.15)
NHL & other LHT	C82–85, C88, C90, C96	18.7	22	1.23 (0.77–1.86)	8.0	12	1.51 (0.78–2.64)
Leukaemias	C91–95	16.0	33	2.17 (1.49–3.05)	6.0	8	1.35 (0.58–2.66)
Other		35.4	71	2.00 (1.57–2.53)	12.7	20	1.58 (0.96–2.43)
Cancers by aetiology							
Alcohol-related	C01–15, C18–22, C32, C50	131.7	316	2.40 (2.14–2.68)	79.7	76	0.95 (0.75–1.19)
Smoking-related	C01–16, C18–22, C25, C30–32, C34, C53, C56, C64–68	257.0	502	1.95 (1.79–2.13)	91.8	134	1.46 (1.22–1.73)
Haematological	C81–85, C88, C90–96	35.2	61	1.73 (1.33–2.23)	14.9	22	1.48 (0.93–2.24)
Hormone-related	C18–21, C50, C54–56, C61, C62, C73	276.8	263	0.95 (0.84–1.07)	119.8	98	0.82 (0.66–0.997)

*Exp* expected cases, *Obs* observed cases, *SIR* standardized incidence ratio, *CI* confidence interval, *NMS* non-melanoma skin, *HL* Hodgkin lymphoma, *NHL* Non-Hodgkin lymphoma, *LHT* lympho-, haematopoietic tissue

## Discussion

This study, based on a large retrospective cohort, has found a higher than expected risk of cancers of most types in men and women with tuberculosis. Increased risks of tobacco-related cancers, alcohol-related cancers and haematological cancers were observed. The risk of hormone-related cancers and melanoma was lower. Risk remained increased for ≥5 years for most smoking- and alcohol-related cancers, cancer of cervix uteri and leukaemias.

Our data on the 1.8-fold increased risk of overall cancer and 1.4-fold increased risk of non-pulmonary cancer among tuberculosis patients are in line with results from previous studies and reviews [4, 9, 22, 23].^.^ In a Danish nationwide cohort study, comparing cancer risk in tuberculosis patients and the general population, the SIR for all cancers was 1.52, 95% CI: 1.45–1.59, whereas for non-pulmonary cancer it was 1.29, 95% CI: 1.22–1.36 [4]. Tuberculosis infection was also associated with increased long-term risk of tobacco-related and haematological malignancies. The incidence of cancer was also increased in the Taiwan Latent Tuberculosis Infection Cohort [8] and in the Estonian population-based cohort where the standardized mortality ratio for all cancers was 2.85, 95% CI: 2.17–3.68 for men and 3.80, 95% CI: 2.22–6.09 for women [22]. Another population-based study in Taiwan found that after a tuberculosis diagnosis the risk was 2.1-fold for total cancer and 1.7-fold for non-pulmonary cancer [9].

Several studies have reported an increased risk of Hodgkin or non-Hodgkin lymphoma in individuals with a history of tuberculosis [9–12]. In addition, significantly elevated long-term risks for leukaemia were observed [4, 9]. Similarly, the elevated SIRs for Hodgkin lymphoma as well as leukaemia were found in our study. Tuberculosis was associated with markedly increased long-term risk of cervical cancer in our study. Our analysis also revealed reduced risk of melanoma among patients with tuberculosis, in contrast with Taiwanese study results, where melanoma risk among men was significantly increased, particularly during <1 year after tuberculosis diagnosis [9]. Our data are in agreement with case-control study performed by Kölmel KF et al. in six European countries and Israel, where significantly lower Odds ratios of melanoma were found for pulmonary tuberculosis, as well as other infections and BCG vaccination [13].

The mechanism underlying the increase of the long-term risk of cancer following tuberculosis is still not clear. Certain infectious episodes including tuberculosis may potentially trigger the development of Hodgkin lymphoma [12]. Previous studies have also supported an underlying immune deficiency many years prior to cancer diagnosis as a primary phenomenon for haematological cancers [12, 24]. Tuberculosis may induce a prolonged inflammatory response hence, inflammation may explain the increased long-term cancer risk. Occurrence of cervical cancer after tuberculosis has previously been reported rarely. There is a possibility that patients with human papilloma virus infection have a higher chance to encounter tuberculosis. In addition, it has been suggested that the *Mycobacterium tuberculosis* infection mediates down regulation of dendritic cells function and thereby facilitates the suppression of the host’s immunity against infectious pathogens and cervical cancer [25]. It has also been suggested that diverse microbial stimuli induce a defence against cancer: both vaccinations and previous episodes of having a severe infectious disease may induce a protective mechanism, possibly an infection-related Th1-cell activation that prevents the development of melanoma [13, 15]. The long-term increased risk may also be due to certain lifestyle factors, such as smoking or alcohol abuse that are strong risk factors for both tuberculosis and cancer [7, 14, 26–31]. E.g., most people with tuberculosis were tobacco smokers (64%) or heavy alcohol users (35%) in our study and we observed elevated all-term SIRs for smoking- or alcohol-related cancers, thus it is plausible that tobacco smoking and alcohol intake are important shared risk factors. Finally, malnutrition is a risk factor for tuberculosis and low body mass index (BMI) is prevalent in tuberculosis patients [32, 33]. There is substantial evidence that obesity increases the risks of breast, endometrial, ovarian cancer, malignant melanoma as well as some other cancers; its influence is most likely mediated through hormonal mechanisms [20]. Low body weight could possibly explain a reduced risk of hormone-related cancers or melanoma in the present study.

The strengths of our study include the use of nationwide population-based registers and the cohort setting which reduced selection bias at baseline. The study also has limitations. There was no appropriate control group to compare the incidence of cancer. It is possible, that patients with tuberculosis differ from the general population with respect to lifestyle factors, like use of tobacco or alcohol. As these factors have been found to be risk factors for various cancer types, confounding by them is likely [27, 28, 34]. In addition, it is possible that the study cohort included individuals with cancer, because tumours may have been interpreted as tuberculosis lesion prior to cancer diagnosis. There is also a potential for bias due to reverse causality, if occult cancer caused a weakening of immunity and malnutrition, resulting in *Mycobacterium tuberculosis* infection or reactivation [35]. Thus, tuberculosis may become clinically apparent earlier than cancer, even though cancer preceded tuberculosis reactivation. Bias from closer medical surveillance received by individuals with tuberculosis than healthy individuals is likely to have had an effect, especially within the first year. However, a reduced risk for melanoma, colon, breast, prostate and thyroid cancer suggests that reverse causality or higher diagnostic activity are unlikely to play major role.

## Conclusions

The overall and non-pulmonary cancer risk was elevated in a cohort of tuberculosis patients in relation to the general Lithuanian population. High risks were observed for smoking- and alcohol-related cancers, haematological cancers and cancer of cervix uteri. In addition, we found reduced risk for hormone-related cancers and melanoma. This retrospective study provides suggestion that tuberculosis may be associated with a risk of some non-pulmonary cancers; however, the possibility of confounding effects cannot be completely ruled out. Further studies are needed to determine the role of potential underlying mechanisms in non-pulmonary cancer development among tuberculosis patients. The recommendation for the awareness of this association among physicians remains warranted.

## Abbreviations

95% CI: 95% Confidence Interval
BMI: Body mass index
ICD-10: International Statistical Classification of Diseases, 10th Revision
LCR: Lithuanian Cancer Registry
SIR: Standardized incidence ratio
WHO: World Health Organization
